# Ovarian traits, spawning pattern and season, length at first maturity, and batch fecundity of *Caragobius urolepis* (Bleeker, 1852) caught from the Vietnamese Mekong Delta

**DOI:** 10.1016/j.heliyon.2024.e39281

**Published:** 2024-10-11

**Authors:** Phuc Le Hoang Nguyen, Lam Thi Thao Vo, Ly Thi Cam Tran, Thoai Kim Nguyen, Thu Thi Anh Phan, Quang Minh Dinh

**Affiliations:** Department of Biology, School of Education, Can Tho University, Viet Nam

**Keywords:** *Caragobius urolepis*, Fecundity, Length at sexual maturity, Mekong Delta, Multiple spawner, Oocyte

## Abstract

*Caragobius urolepis* has a wide range of distribution, from brackish to freshwater water, ranging from India to the Philippines and Fiji, including the Vietnamese Mekong Delta (VMD), where it lives mainly in the coastal regions and plays a vital role in food supply. Our preliminary observation showed that its population tended to decline, but little is known about this species. This study, therefore, was conducted to provide data on the ovarian structure, spawning pattern and season, length at first maturity, and batch fecundity of this fish. The study of 306 specimens caught with trawl nets from April 2022 to March 2023 in two coastal areas of VMD—Dong Hai, Bac Lieu (DHBL), and Dam Doi, Ca Mau (DDCM)—showed that this goby spawned more than once because the oocytes in stage IV ovary showed different stages of development. It could release eggs monthly during a 12-month study, with a peak in July–September since stage IV ovaries were found monthly during the study period and the GSI exhibited a high value in July–September. The fish length at first maturity and batch fecundity varied with the site, as they were 18.8 cm and 3,757–9,187 (6,142 ± 707 SE eggs/female) in DDCM and 20.5 cm and 3,760–11,118 (5,634 ± 750 SE eggs/female) in DHBL. The findings provide valuable foundations for exploiting their resources and serve as a basis for studying artificial reproduction. It is suggested to avoid catching these fish species during the breeding season to ensure sustainable exploitation in the future.

## 1 Introduction

The genus *Caragobius*, belonging to the family Gobiidae, comprises two distinct species, namely *Caragobius rubristriatus* and *Caragobius urolepis* [1]. The former species exhibits a limited distribution range and is primarily found in northern Australia, from the Prince Regent River to the Gulf of Carpentaria. Conversely, the latter species is observed in a broader geographical range, extending from eastern India to Fiji. *Caragobius urolepis,* also known as scaleless worm goby, is widespread throughout the Oceania region, encompassing areas such as South Africa, Indonesia, China, and the Ryukyu Islands of Japan, albeit with some degree of spatial heterogeneity [[2], [3], [4]]. *Caragobius urolepis* is found in the northern region. They usually perch in estuaries and freshwater [5]. Masuda et al. [6] indicated that *Caragobius urolepis* prefers soft mud bottoms in estuaries. Furthermore, this species constructed burrows within the muddy substrate and feeds on a diet consisting of zooplankton and crustaceans [5].

*Caragobius* species exhibit a preference for occupying submerged areas with considerable depth as well as utilizing diverse sheltering structures. Within the Mekong Delta in Vietnam, a specific species known as *Caragobius urolepis* [7], commonly called the scaleless worm goby, has been taxonomically identified. According to the research conducted by Diep et al. [8], this species is recognized as part of the Gobiidae family and is associated with the Mekong Delta region. In the Mekong Delta (Vietnam), *Caragobius urolepis*, according to Diep et al. [8], has been identified as belonging to 12 genera and 16 species within the Gobiidae family. These organisms primarily inhabit brackish water areas and estuaries within the Mekong Delta ecosystem. Within the contextual framework of Vietnam, *Caragobius urolepis* assumes notable significance across various realms, including culture, economy, and biodiversity.

*Caragobius urolepis* is vital to Vietnam's culture, economy, and biodiversity. This fish species is an essential food source and an integral component of Vietnamese cultural activities and traditions owing to its high nutritional content and economic value [9]. In light of the intricate nature of its habitat and the heterogeneous distribution patterns, *Caragobius urolepis* has assumed a pivotal role in certain regions' agricultural sectors as an indispensable component. However, based on the preliminary findings of this research, it became evident that there has been a discernible decline in the quantity of fish being captured in recent times. Hence, in an effort to address challenges and maintain the sustainability of its fisheries, the Vietnamese government has approved a national development program focused on promoting efficient and environmentally responsible fishing practices. This program, which covers the period from 2022 to 2025 with guidance extending to 2030, emphasizes the implementation of a Total Allowable Catch (TAC) system and a quota-based management approach to regulate fishing activities [10].

*Caragobius urolepis* has been the subject of numerous studies conducted by scientists worldwide, as documented in Table 1. Among these studies, a specific investigation by Nguyen et al. [11] focused on exploring the testicular characteristics of male *Caragobius urolepis*. This study provided valuable insights into the reproductive biology specific to male individuals. However, the reproductive biology of female *Caragobius urolepis* remains unexplored, as no research has been conducted in this area. Therefore, this study represents the first attempt to investigate various reproductive biology aspects, including ovarian traits, spawning patterns and seasons, length at first maturity, and batch fecundity, within the ecosystem of the Mekong Delta region. The main objective of this research is to provide comprehensive information on the reproductive biology of *Caragobius urolepis*. Moreover, conducting this study is expected to yield valuable insights and contribute to a deeper understanding of reproductive biology, enhancing the knowledge base in this field.

**Table 1:** Distribution, ecology, and reproduction for *Caragobius urolepis*.

Waterbody	Aspects	References
Japan	Ecology	[6]
	Distribution	[2,[3], [4]]
Indo-Pacific (India, Thailand, Indonesia, the Philipines, Taiwan, Japan, Fiji)	Species composition	[1]
Taiwan	Ecology	[5]
Mekong Delta region	Taxonomical identification	[7]
Soc Trang province	Species composition	[8]
Ca Mau province	Ecology	[12]
China	Ecology	[5]
Bac Lieu, Ca Mau	Vietnam's law	[10]
	Species' value	[9]
Bac Lieu, Ca Mau	Male reproductive traits	[11]

## 2 Materials and methods

**Fig. 1:**
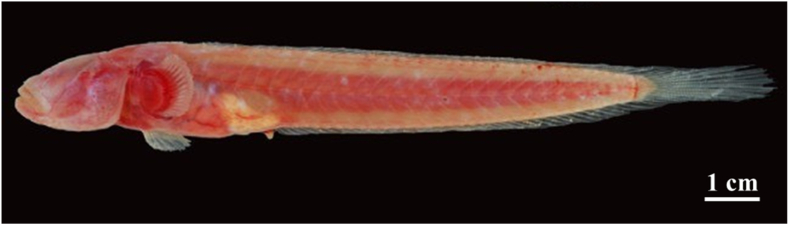
*Caragobius urolepis* at two sites.

**Fig. 2:**
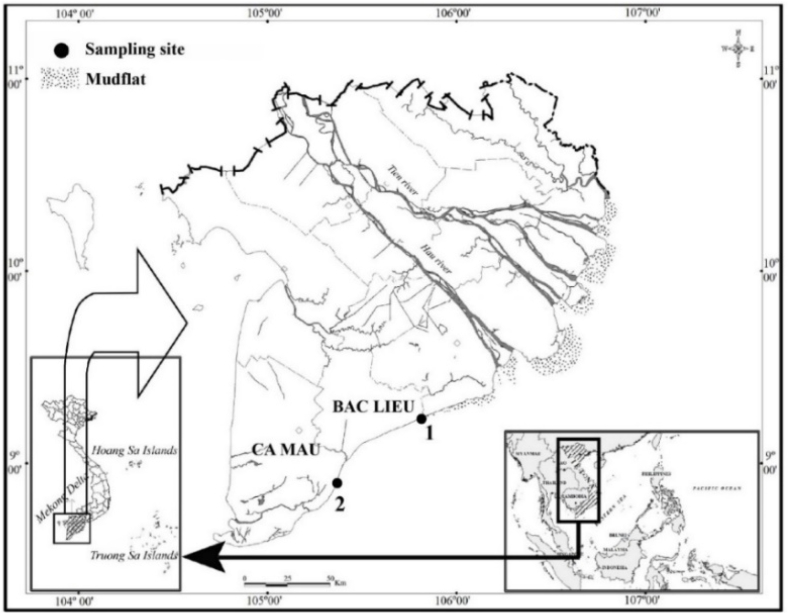
Map of the Mekong Delta showing the sampling locations (1: Dong Hai, Bac Lieu; 2: Dam Doi, Ca Mau; Source: modified from Dinh [14]).

The *Caragobius urolepis* (Fig. 1) was captured directly by hand and indirectly with trawl nets by local fishermen, utilizing mesh sizes of 1.0 cm. The sample collection process was scheduled to occur monthly from April 2022 to March 2023 at two distinct locations: Dong Hai in Bac Lieu (DHBL) and Dam Doi in Ca Mau (DDCM) (Fig. 2). In the study, a total of 306 samples were included. Among the collected fish specimens, DDCM constituted a significantly higher proportion, with 165 individuals, while DHBL comprised 141 (Table 2). In the Bac Lieu region, salinity levels were recorded at an average of 21.6 ± 6.2 ‰, as determined by Truong et al. [13]. Conversely, the average salinity in Ca Mau was notably lower, with a value of only 4.7 ± 1.7 ‰, as Le et al. [12] reported. The prevailing average temperature within the studied areas ranged between 23 and 28 °C.

**Table 2:** Number of female fish per month.

Sites		
Months	Dong Hai, Bac Lieu	Dam Doi, Ca Mau
04/2022	14	15
05/2022	13	12
06/2022	12	18
07/2022	11	12
08/2022	16	13
09/2022	16	11
10/2022	10	16
11/2022	06	14
12/2022	09	15
01/2023	12	13
02/2023	13	11
03/2023	09	15
**Total**	**141**	**165**

Fish specimens were collected non-selectively, encompassing various developmental stages, throughout the year at the two designated research locations.

Following the collection of each sample at the respective research sites, the specimens were subjected to a washing process and subsequently immersed in a 4 % formalin solution, serving as a preservative, before their transportation to the laboratory. Given the diverse size range of the captured fish, a subsequent classification of the specimens was conducted in the laboratory based on the morphological characteristics of the genital papular. Based on the research undertaken by Nguyen et al. [9], it was observed that one exhibiting an oval shape could be identified as a female specimen. Then, the fish's ovaries were removed and weighed to 0.01 mg. These ovarian samples were subsequently categorized into six distinct stages, employing the methodology established by Dinh et al. [15]. The specimens were preserved before histological analysis by immersing them in 70 % aldehyde. The ovaries were engaged in a 4 % formalin solution prepared by diluting 1 part formalin with nine parts water to conduct the histological examination. This study did not require animal ethics approval, as dead fish were used.

This research combines the double-staining method described by Carleton et al. [16] and the histological method developed by Dinh & Nguyen [17]. The procedure for performing a fixed microscopic specimen of the gonads of fish was utilized. The staining process in this research included sample preparation and fixation, dehydration and impregnation with paraffin, paraffin mold casting, sample cutting, and dyeing samples.

According to the research by Alonso-Fernández et al. [18] and Dinh & Le [19], the gonads of *Caragobius urolepis* were used to figure out when the spawning season was. Additionally, Lloret & Rätz [20] suggested that the gonadosomatic index (GSI) was calculated using the formula GSI = (GW/W) × 100, where GW represented the weight of the gonads and W denoted the total body weight of the fish. The MGSI (modified gonadosomatic index) was calculated by Nikolsky [21] as MGSI (%) = (GW/W-GW) × 100 and DI (Dobriyal index) by Dobriyal et al. [22] as DI = ∛GW.

The batch fecundity was calculated as F = n × G/g [23] (G: the ovarian weight, n: the number of mature ovaries (stage IV), and g: the egg mass in a sub-sample (0.1 mg)). Three tissue sub-samples of 1 mm thick were extracted from each ovary: two samples from each end and one from the mid ovary. Each slice was weighed to the nearest 0.1 mg, and oocytes were separated with tap water and a spear needle in a Petri dish. Then, the mature oocytes were observed and counted using Motic Images Pro Plus 2.0 software [24].

According to Zar [25], the first mature length (L_m_) was calculated as P = 1/(1 + exp[–r(TL – L_m_)]). The L_m_ corresponded to the body length at which 50 % of the fish population reached stage III of gonad maturation. Some researchers, including Dinh et al. [26], found that the spawning season of this fish species could be predicted by looking at how the GSI changed while samples were being collected. This study employed non-linear regression analysis to elucidate the relationships between fecundity and fish size (length and weight). All statistical calculations were performed using SPSS v21 software, with a predetermined significance level of 5 %.

According to Hossain et al. [27] and Hasan et al. [28], the size at sexual maturity was calculated using the following equation of Binohlan & Froese [29]: log(L_m_) = −0.1189 + 0.9157∗log(L_max_).

The Shapiro-Wilk test was used to confirm the normal distribution of GSI [30]. A *t*-test combined with the Levene test for equality of variances was applied to evaluate seasonal changes in GSI when it followed a normal distribution; otherwise, the Mann-Whitney test was used. For each site, if GSI was normally distributed, 1-way ANOVA with Tukey's post hoc test was conducted to assess monthly changes in GSI when variances were equal, but 1-way ANOVA with Tamhane's T2 was utilized if variances were unequal. Conversely, if GSI did not follow a normal distribution, the Kruskal-Wallis test was employed to investigate monthly changes in GSI at each site. The relationship between batch fecundity and fish size (W and TL) was determined using logarithmic regression [31,32].

## 3 Results and discussion

### 3.1 Developmental stages of oocytes

**Fig. 3:**
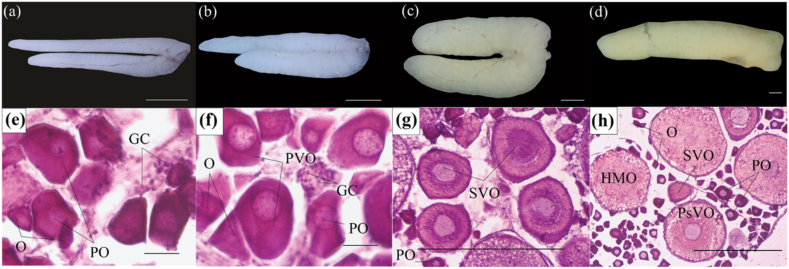
External morphology and histology of ovaries of *Caragobius urolepis*. (a–d: stages I-IV: external ovarian morphology; e–h: stages I–V: ovarian histological structure; GC: germ cells, O: oogonia, PO: primary oocyte, PVO: primary vitellogenic oocytes, SVO: secondary vitellogenic oocytes, PsVO: post vitellogenic oocytes, HMO: hydrated oocytes. Scale bar: 1 mm for external morphology and 50 μm for histology).
Stage II: The ovarian structures exhibited opaque white, elongated, and double-filament characteristics while rapidly increasing width (Fig. 3b). These attributes were observable to the naked eye at this stage. At this stage, specific components could be identified within the ovaries, including O, PO, and primary vitellogenic oocytes (PVO). Among these, PO and PVO occupied the most significant proportion of space within the ovary and alternated in their distribution. Notably, PVO differed from PO because it had a clear nuclear circle inside the nucleus that turned blue-violet when stained with hematoxylin (Fig. 3f).
Stage III: The ovaries exhibited a pale yellow color, remarkable development in width, and a smooth and glossy surface (Fig. 3c). These ovaries took on a nearly spherical shape and gradually loosened their connection, becoming easily separable. They could be observed with the naked eye. At this stage, the ovaries comprised O, PO, and supplementary vitellogenic oocytes (SVO). Among these, SVO predominated, while PO and O were interspersed. The formation of ovule vesicles (containing ovule granules) was initiated within the SVO (Fig. 3g).
Stage IV: The ovarians were elongated and reached near-maximum development in size, occupying a significant portion of the abdominal cavity. These ovaries exhibited a pale yellow color and a smoother, swollen surface. Blood vessels began to appear on the surface. Due to their large size, the oocytes were easily observable to the naked eye and could be readily separated into individual granules (Fig. 3d). Post-vitellogenic oocytes (PsVO) and hydrated oocytes (HMO) were approximately proportionally equal at this stage. The ovary filled almost the entire volume, appearing pink and spherical. The nuclei became condensed, the nuclear membranes underwent involution, and the nuclear nucleoli moved toward the center of the nucleus (Fig. 3h).
Stages V and VI: These two stages were not observed during the study period, suggesting that the fish may have migrated to other spawning sites.

Stage I: The ovarian structures were characterized by immaturity, exhibiting a doubled filamentous shape (Fig. 3a). They appeared milky white, and the ovaries were thin and elongated at this stage. The oocytes comprised several components within stage I, including a small oogonia (O) that occupied a significant portion of the space. The cytoplasm of oogonia appeared dark purple when stained with hematoxylin. Furthermore, there were large-sized primary oocytes (PO) and small-sized germ cells (GC). Notably, the primary oocytes exhibited a central nuclear area that gradually faded towards the periphery and had yet to develop a yolk sac (Fig. 3e).

The histological analysis of the ovary of *Caragobius urolepis* indicated that this species underwent multiple spawning due to the presence of oocytes at different stages during ovary development. These findings were consistent with several studies, including *Mystus mysticetus* [33], *Periophthalmus* *schlosseri* [17], and *Glossogobius sparsipapillus* [34].

### 3.2 Spawning season

**Fig. 4:**
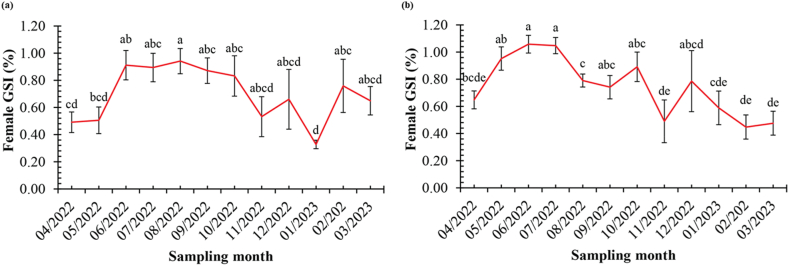
The GSI of *Caragobius urolepis* (a and b represent Dong Hai, Bac Lieu; Dam Doi, Ca Mau; Different letters (a, b, c, and d) denote monthly variations in GSI values at each location).

**Fig. 5:**
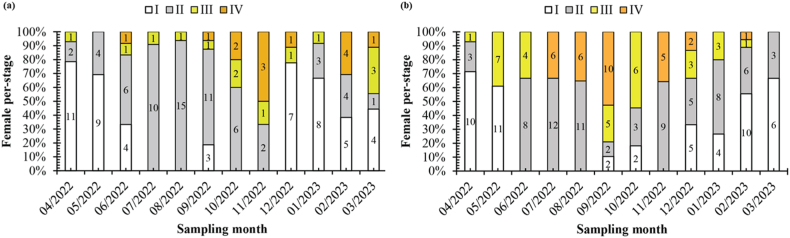
The frequency of occurrence of the ovarian developmental stages of *Caragobius urolepis* (a and b represent Dong Hai, Bac Lieu; Dam Doi, Ca Mau; number in each column; number of individuals).

The spawning season of *Caragobius urolepis* was found by looking at how often the gonads appeared during different stages of oocyte maturation and the GSI. In the DDCM population, the GSI of females (Fig. 4b) exhibited a higher value than the GSI observed in the DHBL population (Fig. 4a). In the DHBL population (Fig. 5a), a mature ovary was seen from June 2022 to March 2023. It kept showing up from September to November 2022, with most sightings happening in October, November, and February. Conversely, in the DDCM population (Fig. 5b), the analysis demonstrated that the ovary in the mature stage started to appear from July 2022 to September 2022, with February 2023 marking the highest occurrence. Additionally, the frequency of gonad occurrence revealed that stage IV of oocyte maturation manifested continuously between July and September, with the highest frequency observed in September and November.

The analysis of Fig. 5 revealed the fluctuations of the GSI of *Caragobius urolepis* ovaries over 12 months. In DHBL (Fig. 5a), the chart depicted a rapid increase in the ovary GSI from May to June. Subsequently, the index reached its highest level from June to August, followed by a gradual decline from August to October, although it remained relatively elevated. A distinct decrease was observed between October and November, followed by an increase in December and subsequent fluctuations, with a declining trend in the following months until February 2023. In DDCM (Fig. 5b), the GSI exhibited changes throughout the study duration. Specifically, an evident increase in this index was observed from April to July. However, a slight decrease was observed after July, followed by a subsequent rise in October. A sharp decline was observed in November, with an increase in December. A similar trend was found in mGSI and DI: those were 0.05–2.23 mGSI and 0.14–0.90 DI in DHBL and 0.03–2.86 mGSI and 0.14–0.83 DI in DDCM.

In this research, DDCM had a salinity of 4.7 ± 1.7 ‰ lower than DHBL (21.6 ± 6.2 ‰), and the GSI at DDCM was higher than at DHBL. It seemed that brackish water, which had lower salinity index, was favorable for this species. On the same line, salinity greatly influenced gamete development and the spawning of *Crassostrea madrasensis* and *Crassostrea virginica* [[35], [36]]. Moreover, Lenz & Boehs [37] stated that the decrease in salinity due to heavy rainfall might have triggered gamete release in *Crassostrea rhizophorae* in Camamu Bay, Brazil. This also was consistent with the research of Gomes et al. [38], who found that the salinity influenced the reproductive development of *Crassostrea gigas*. In the same light, *Mystus albolineatus* was observed to successfully reproduce under lower salinity conditions compared to other fish species. This suggested that the water's salinity levels had an impact on the development of the eggs and the hatching process for this particular species.

It was clear that the fish started reproducing during the wet season, which runs from June to October, because of the GSI and the number of times the gonads were seen. The fish put most of their efforts into reproducing during the busiest months, like July–September and November. These results exhibited similarities with other species within the same family. For instance, Truong et al. [13] reported that *Pseudapocryptes elongatus* (Cuvier, 1816) demonstrated a spawning season during the rainy months from May to November, synchronizing with the monthly high tides on the 15th and 30th of the lunar calendar and exhibited high population densities from June to September in areas abundant with coastal mangrove forests. Similarly, in another fish species belonging to the Gobiidae family, Dinh et al. [39] observed comparable breeding patterns in the gold-spotted mudskipper *Periophthalmus chrysospilos* within the same ecosystem as *Caragobius urolepis*, with a peak spawning season during the rainy months from July to October. Moreover, *Mystus mysticetus*, belonging to the Bagridae family, exhibited the highest breeding activity during the wetseason, particularly peaking in June–October, as Vo et al. [33] reported. Additionally, Nguyen et al. [34] indicated that *Glossogobius sparsipapillus* also reproduced during the wetseason, specifically from July to September, in a continuous manner. These collective studies provided insights into the breeding seasons of various fish species within the same family and highlighted the influence of rainfall patterns and the synchronization of reproductive activities with seasonal environmental conditions.

### 3.3 Length at the first maturity

**Fig. 6:**
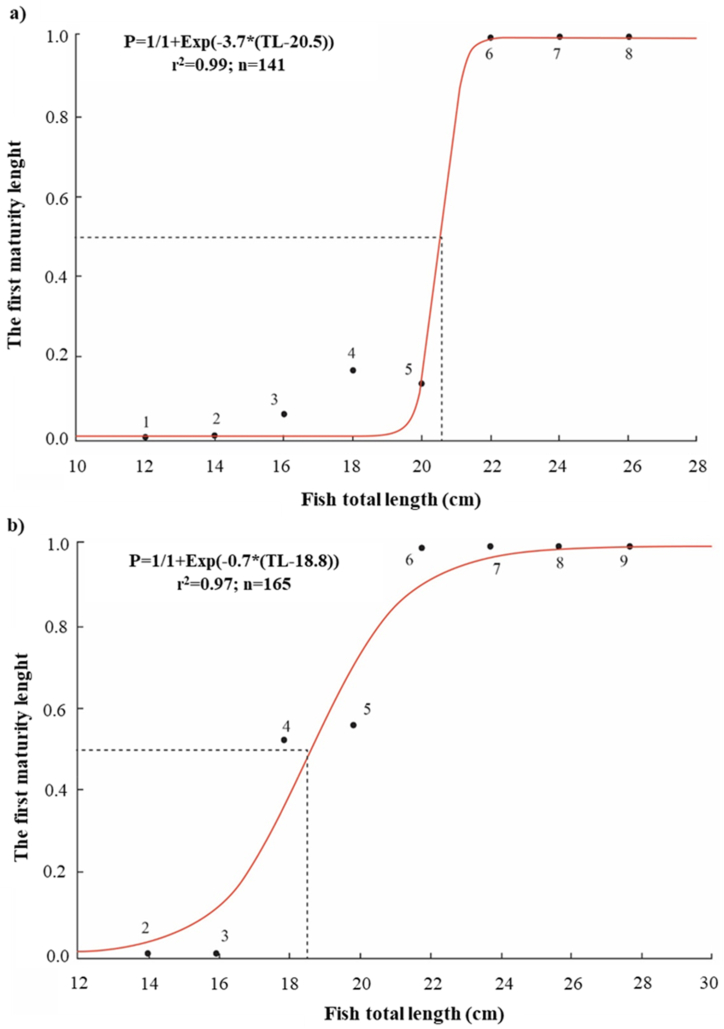
The first maturity length of *Caragobius urolepis* females was determined as follows: (a) in Dong Hai, Bac Lieu, and (b) in Dam Doi, Ca Mau (The dashed square was the trendline of the length at the first mature equation by plotting the proportion of females with stage III of the ovaries or later in each fish size (TL); The number of the exponential curve meant the total of individual fish was used).

The first mature length (L_m_) —the size at which 50 % of individuals reached the maturity stage— of *Caragobius urolepis* was presented in Fig. 6.

The findings related to the first mature length were based on 306 specimens. In DHBL, female *Caragobius urolepis* exhibited the first maturity length of up to 20.5 cm (Fig. 6a), while in DDCM, the first maturity length of females was recorded at 18.8 cm (Fig. 6b). According to the provided parameters, it was apparent that the attainment of the first mature length in *Caragobius urolepis* occurred at a greater length in DHBL than in DDCM. Consequently, it could be deduced that *Caragobius urolepis* displayed a propensity toward faster growth rates in DHBL than in DDCM. The difference was probably because the salinity concentration in DDCM was higher than in DHBL. Furthermore, it was noteworthy that *Caragobius urolepis* exhibited superior growth performance in brackish water environments. Hence, it could be deduced that the variations observed in the first maturity length of *Caragobius urolepis* was intricately influenced by the combined effects of biotic and abiotic factors specific to each sampling location. This finding was in light of *Glossogobius sparsipapillus* [34] and *Periophthalmodon septemradiatus* [40]. According to Dinh et al. [41], the length growth of *Ellochelon vaigiensis* was significantly higher in a diverse mangrove ecosystem with salinity levels ranging from 10.4 ‰ to 16.7 ‰. Similarly, Otieno et al. [42] observed that changes in food availability, water quality, as well as fluctuations in water level and temperature played a crucial role in determining the size of fish.

Following the results of the first mature length, it was concluded that *Caragobius urolepis* was well-adapted to environments with salty water, indicating that it could do well there. This finding concurred with previous research conducted by Kottelat et al. [2], which posited that Caragobius urolepis exhibited robust growth and development in brackish water environments. This assertion was further supported by Tran [43], investigating *Pseudapocryptes elongatus*, and by Nguyen et al. [34], studying *Glossogobius sparsipapillus*. Additionally, this study's outcomes aligned with the conclusions drawn by Wootton [44], who contended that the first maturity length of fish was subject to variation based on local and global environmental factors. Furthermore, Fouda et al. [45] reported that *Pomatoschistus marmoratus* attained 2.4 cm and 2.7 cm lengths in the Suez Canal and Maguio Lagoon, respectively. Collectively, these investigations underscored the notion that fish species' growth and maturation processes, including those of *Caragobius urolepis*, were influenced by a combination of environmental factors specific to their respective local habitats and broader ecological considerations.

### 3.4 Batch fecundity

The batch fecundity of *Caragobius urolepis* within the DHBL population ranged from 3,757 to 9,187 eggs/female, with an average of 5,634 ± 750 SE. Conversely, the fish sample obtained from DDCM displayed a comparatively higher but statistically non-significant reproductive capacity, ranging from 3,760 to 11,118 eggs per female, with an average of 6,142 ± 702 SE. This subtle difference provided evidence that variations in salinity, pH, and environmental temperature did not exert discernible effects on the batch fecundity of *Caragobius urolepis*.

**Fig. 7:**
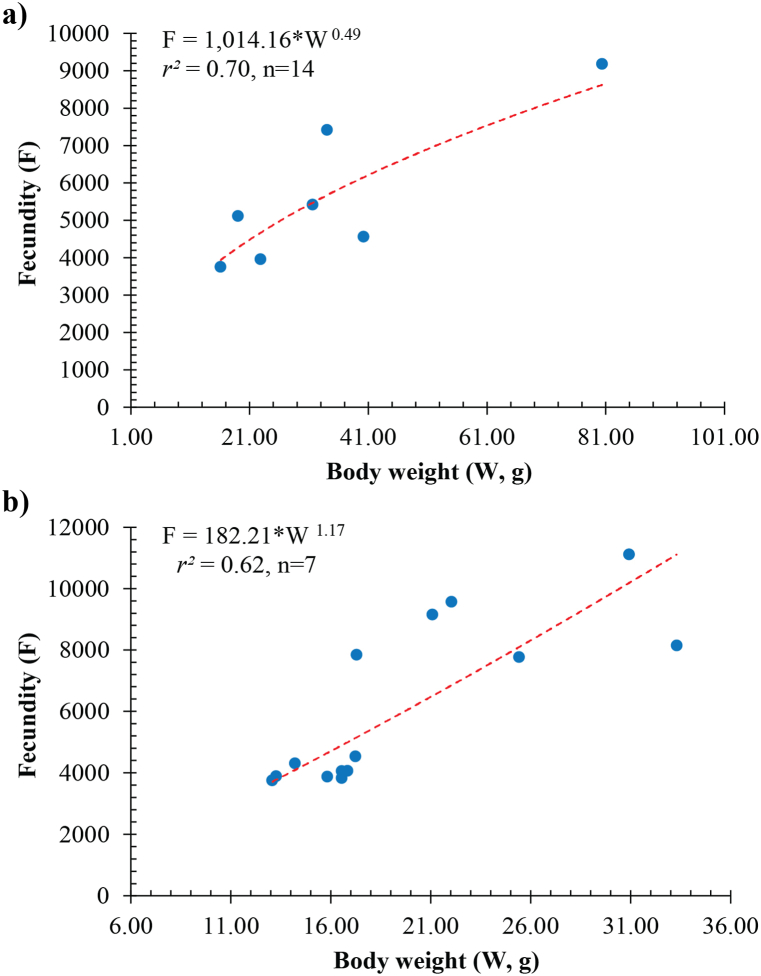
Relationships between the batch fecundity (F) and body weight (W) of *Caragobius urolepis* at Dam Doi, Ca Mau (a) and Dong Hai, Bac Lieu (b).

**Fig. 8:**
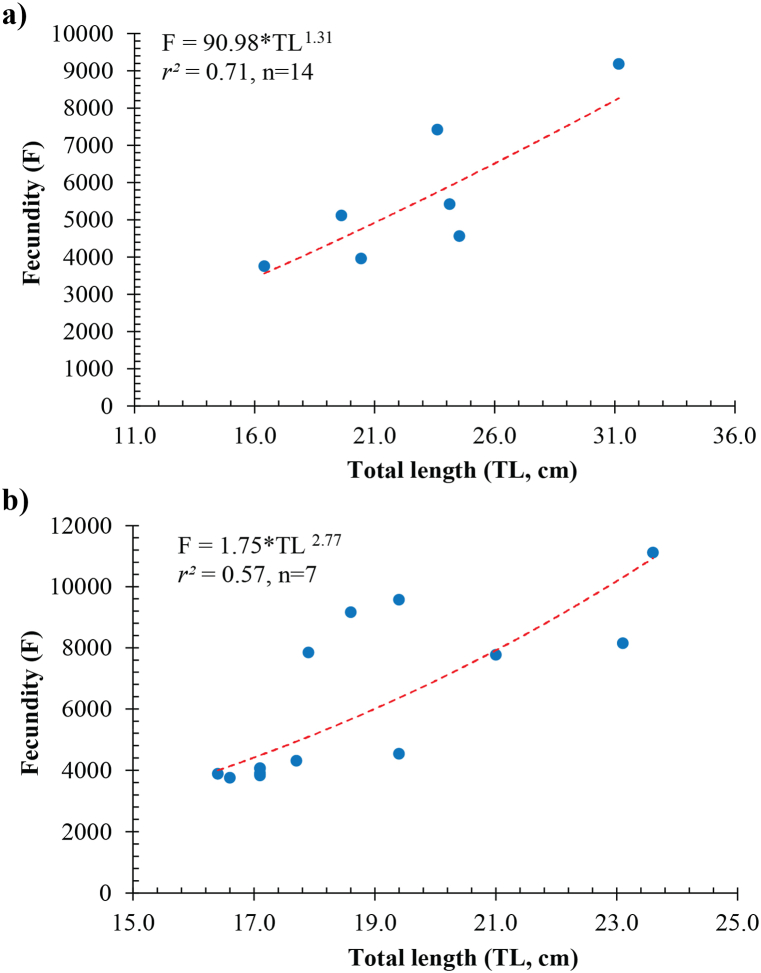
Relationships between the batch fecundity (F) and total length (TL) of *Caragobius urolepis* at Dam Doi, Ca Mau (a) and Dong Hai, Bac Lieu (b).

Based on the regression analysis (Fig. 7, Fig. 8), a significant association existed between the fecundity of *Caragobius urolepis* and their weight (Fig. 7a in DBBL and Fig. 7b in DDCM) and length (Fig. 8a in DBBL and Fig. 8b in DDCM). It could be posited that the fish's weight and length strongly correlated with their fecundity, indicating that these morphological characteristics played a crucial role in determining the batch fecundity of *Caragobius urolepis*.

*Caragobius urolepis* in the present study had a lower batch fecundity than *Boleophthalmus boddarti* (ranging from 9,800 to 33,800 eggs per female) of Dinh [46]. The batch fecundity of this species was higher than that of *Neogobius melanostomus*, as seen in studies by Macinnis & Corkum [47] (ranging from 84 to 600 eggs per female) and Caputo et al. [48] on *Crystallogobius linearis* (ranging from 200 to 700 eggs per female).

## 4 Conclusions

*Caragobius urolepis* exhibited a multi-spawning pattern, with peaks in July, September, and November. The batch fecundity of *Caragobius urolepis* was 3,760 to 11,118 eggs per female. In order to promote the sustainable exploitation and long-term economic viability of *Caragobius urolepis*, fishermen should adhere to resource management regulations that include limitations on the capture of fish below the first mature length (L_m_) and the avoidance of fishing during the spawning period. Furthermore, establishing conservation areas might be imperative as it could facilitate resource recovery and ecological product preservation, ultimately contributing to maintaining marine ecosystem biodiversity. These measures collectively aimed to ensure the prudent management of *Caragobius urolepis* populations and conserve their environmental integrity.

## CRediT authorship contribution statement

**Phuc Le Hoang Nguyen:** Writing – review & editing, Writing – original draft, Methodology, Investigation, Funding acquisition. **Lam Thi Thao Vo:** Writing – review & editing, Writing – original draft, Methodology, Investigation, Funding acquisition. **Ly Thi Cam Tran:** Writing – review & editing, Writing – original draft, Methodology, Investigation, Funding acquisition. **Thoai Kim Nguyen:** Writing – review & editing, Writing – original draft, Methodology, Investigation, Funding acquisition. **Thu Thi Anh Phan:** Writing – review & editing, Writing – original draft, Methodology, Investigation, Funding acquisition. **Quang Minh Dinh:** Writing – review & editing, Writing – original draft, Supervision, Methodology, Conceptualization.

## Data availability statement

Data will be made available on request.

## Declaration of competing interest

The authors declare that there is no conflict of interest regarding the publication of this article.

The manuscript is authentic and is not currently submitted or reviewed or published in any other journal.
